# *Helicobacter pylori* pathogen inhibits cellular responses to oncogenic stress and apoptosis

**DOI:** 10.1371/journal.ppat.1010628

**Published:** 2022-06-29

**Authors:** Manikandan Palrasu, Elena Zaika, Kodisundaram Paulrasu, Ravindran Caspa Gokulan, Giovanni Suarez, Jianwen Que, Wael El-Rifai, Richard M. Peek, Monica Garcia-Buitrago, Alexander I. Zaika

**Affiliations:** 1 Department of Surgery, Miller School of Medicine, University of Miami, Miami, Florida, United States of America; 2 Department of Medicine, Vanderbilt University, Nashville, Tennessee, United States of America; 3 Department of Medicine, Columbia University Medical Center, New York, New York, United States of America; 4 Department of Veterans Affairs, Miami VA Healthcare System, Miami, Florida, United States of America; 5 Department of Pathology, University of Miami Miller School of Medicine, Miami, Florida, United States of America; University of Illinois, UNITED STATES

## Abstract

*Helicobacter pylori* (*H*. *pylori*) is a common gastric pathogen that infects approximately half of the world’s population. Infection with *H*. *pylori* can lead to diverse pathological conditions, including chronic gastritis, peptic ulcer disease, and cancer. The latter is the most severe consequence of *H*. *pylori* infection. According to epidemiological studies, gastric infection with *H*. *pylori* is the strongest known risk factor for non-cardia gastric cancer (GC), which remains one of the leading causes of cancer-related deaths worldwide. However, it still remains to be poorly understood how host-microbe interactions result in cancer development in the human stomach. Here we focus on the *H*. *pylori* bacterial factors that affect the host ubiquitin proteasome system. We investigated E3 ubiquitin ligases SIVA1 and ULF that regulate p14ARF (p19ARF in mice) tumor suppressor. ARF plays a key role in regulation of the oncogenic stress response and is frequently inhibited during GC progression. Expression of ARF, SIVA1 and ULF proteins were investigated in gastroids, *H*. *pylori*-infected mice and human gastric tissues. The role of the *H*. *pylori* type IV secretion system was assessed using various *H*. *pylori* isogenic mutants. Our studies demonstrated that *H*. *pylori* infection results in induction of ULF, decrease in SIVA1 protein levels, and subsequent ubiquitination and degradation of p14ARF tumor suppressor. Bacterial CagA protein was found to sequentially bind to SIVA1 and ULF proteins. This process is regulated by CagA protein phosphorylation at the EPIYA motifs. Downregulation of ARF protein leads to inhibition of cellular apoptosis and oncogenic stress response that may promote gastric carcinogenesis.

## Author summary

Stomach infection with *Helicobacter pylori* bacteria is considered to be one of the strongest known risk factors for gastric cancer. Understanding mechanisms regulating carcinogenic interactions between bacteria and human cells is important for development of new effective therapies against gastric cancer. In this work, we report how bacterial protein CagA alters intracellular regulation inside human cells, causing inhibition of tumor suppression mechanism termed the Oncogenic Stress Response controlled by human protein p14ARF. Our studies revealed that bacterial protein CagA interacts with two human proteins SIVA1 and ULF, resulting in activation of ULF and rapid degradation of SIVA1 and p14ARF, which regulate cell death of tumorous cells.

## Introduction

*Helicobacter pylori* is a Gram-negative, microaerophilic bacterium that evolved to survive in the hostile acidic environment of the human stomach and successfully colonize the gastric mucosa. Chronic infection with this pathogen is considered the principal cause of gastric cancer, with almost all non-cardia gastric cancer (GC) cases attributed to this bacterial infection [1]. Gastric carcinogenesis associated with *H*. *pylori* infection is determined by complex and still not well understood interactions between bacteria and human cells [2]. Previous studies have shown that one of the bacterial virulence determinants closely linked to tumorigenesis is CagA protein [3]. The *cagA* gene, which encodes CagA protein, is located at the 3’ end of the *cag* pathogenicity island (*cag* PAI.) The products of the *cag* PAI form type IV secretion system (T4SS) pili that deliver CagA protein inside gastric cells. After translocation, CagA is phosphorylated by host tyrosine kinases at the EPIYA (Glu-Pro-Ile-Tyr-Ala) repeatable motifs located at the carboxy-terminal end of the CagA molecule. CagA is responsible for alteration of multiple signaling pathways and aberrant activation of various oncogenic proteins such as Ras, β-catenin, PI3K, and others [2]. The full spectrum of host interacting partners of CagA protein still remains unclear.

Aberrant activation of oncogenes, which may be caused by bacterial and viral agents, triggers cell protection mechanism termed the oncogenic stress response (OSR). Mechanistically, the OSR functions as a surveillance program that eliminates cells with aberrantly activated oncogenes by inducing their death or permanent cell cycle arrest, thereby preventing carcinogenesis [4,5]. One of the key regulators of the OSR is ARF protein (termed p19ARF in mice and p14ARF in humans), a product of the INK4a/ARF locus. ARF protein induces apoptosis and permanent cell cycle arrest by activating p53 protein, but it also efficiently functions in a p53-independent manner [6]. We have previously reported that ARF increases stability of p53 protein in *H*. *pylori*-infected cells [7]. ARF protein has been implicated in antiviral activity. Some viruses, such as EBV and polyoma virus developed mechanisms counteracting ARF functions [8,9].

Protein levels of p14ARF are regulated at the transcriptional and posttranslational levels. A number of E3 ubiquitin ligases, MKRN1, ULF, and SIVA1 has been implicated in regulation of ARF protein degradation and its activity [10–12]. Their dysregulation may have a tumor promoting effect. Oncogenic role of MKRN1 and ULF proteins have been previously proposed [11,13–15]. Contradictory evidence exists regarding the role of SIVA1, as this protein is a potent inducer of extrinsic and intrinsic apoptotic pathways, but, in certain circumstances, may facilitate cancer development [16]. We have recently reported that Siva1 is inhibited by *H*. *pylori* [17].

In this study, we investigated how *H*. *pylori* bacterial factors regulate ARF and the OSR.

## Results

### *H*. *pylori* downregulates p14ARF protein in a strain-specific manner

**Fig 1:**
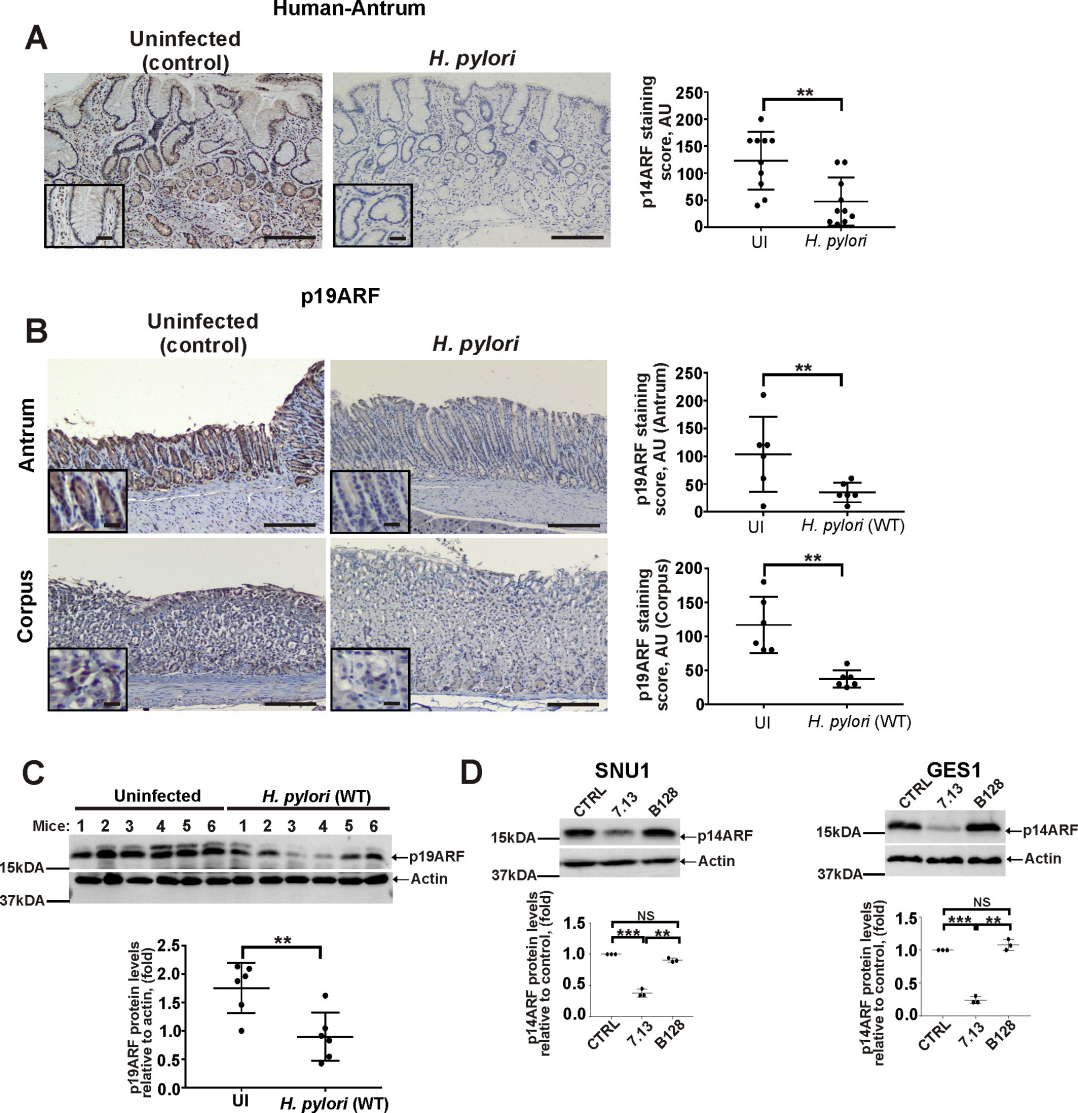
*H*. *pylori* infection leads to downregulation of p14ARF. (A) Representative images showing IHC staining for p14ARF protein in antral gastric biopsies collected from *H*. *pylori*-infected and non-infected patients with active chronic gastritis. The graph panel shows IHC scores for p14ARF (n = 10/group). (B) Representative images of IHC staining for p19ARF protein in the corpus and the antrum of *H*. *pylori*-infected and uninfected mice. The graph panel shows IHC scores for p19ARF (n = 6/group). (C) The same as (B), but gastric tissue homogenates from mice were analyze for p19ARF protein expression by Western blotting. Bottom panel shows the densitometric analysis of p19ARF protein expression. (D) Analysis of p14ARF protein expression in SNU1 and GES1 cells co-cultured with *H*. *pylori* strains 7.13 or B128 for 6hrs. The graph panels represent densitometric analyses of p14ARF protein expression. Expression of p14ARF protein in uninfected cells was arbitrarily set at 1. Data in A, B, and C were analyzed using unpaired 2-tailed t-test; data in D were analyzed using 1-way ANOVA followed by Tukey’s multiple comparison test. Data are displayed as mean ± SD (n = 3). Insets in (A) and (B) show the magnified views (×40). Scale bars: 50 μm.

To investigate regulation of the OSR by *H*. *pylori*, we first analyzed expression of p14ARF protein, which plays a central role in the OSR, using immunohistochemistry (IHC) in gastric biopsies collected from *H*. *pylori-*infected patients and uninfected control subjects (20 patients; 10/group) who were diagnosed with active chronic gastritis. Our analysis revealed that *H*. *pylori* infection leads to significant downregulation of p14ARF in gastric epithelial cells (Fig 1A). p14ARF primarily showed nucleocytoplasmic staining (S1A Fig). Significant downregulation of p19ARF protein, a murine homolog of human p14ARF, was also found in the murine stomach infected with *H*. *pylori*. C57BL/6 mice were gavaged with *H*. *pylori* strain PMSS1, which expresses a functional CagA and efficiently colonizes the murine mucosa [18], while control group received Brucella broth (n = 6/group). ARF expression was assessed in the gastric antrum and corpus 10 days after *H*. *pylori* inoculation, using IHC (Fig 1B). To validate IHC analyses, murine gastric tissue homogenates were also analyzed using Western blotting (Fig 1C).

To explore how p14ARF is affected by *H*. *pylori* in a more controlled environment, we analyzed expression of p14ARF in SNU1 and GES1 human gastric epithelial cells that express high levels of ARF protein. Cells were co-cultured with clinical isolate B128 and its cancerogenic derivative 7.13 for 6hrs. The latter strain, but not the former, strongly activates various cellular oncogenic pathways and causes premalignant and malignant gastric lesions in different animal models [19,20]. Tumorigenic strain 7.13 was found to strongly downregulate p14ARF while significantly weaker changes were found after infection with the parental strain B128 (Fig 1D).

**Fig 2:**
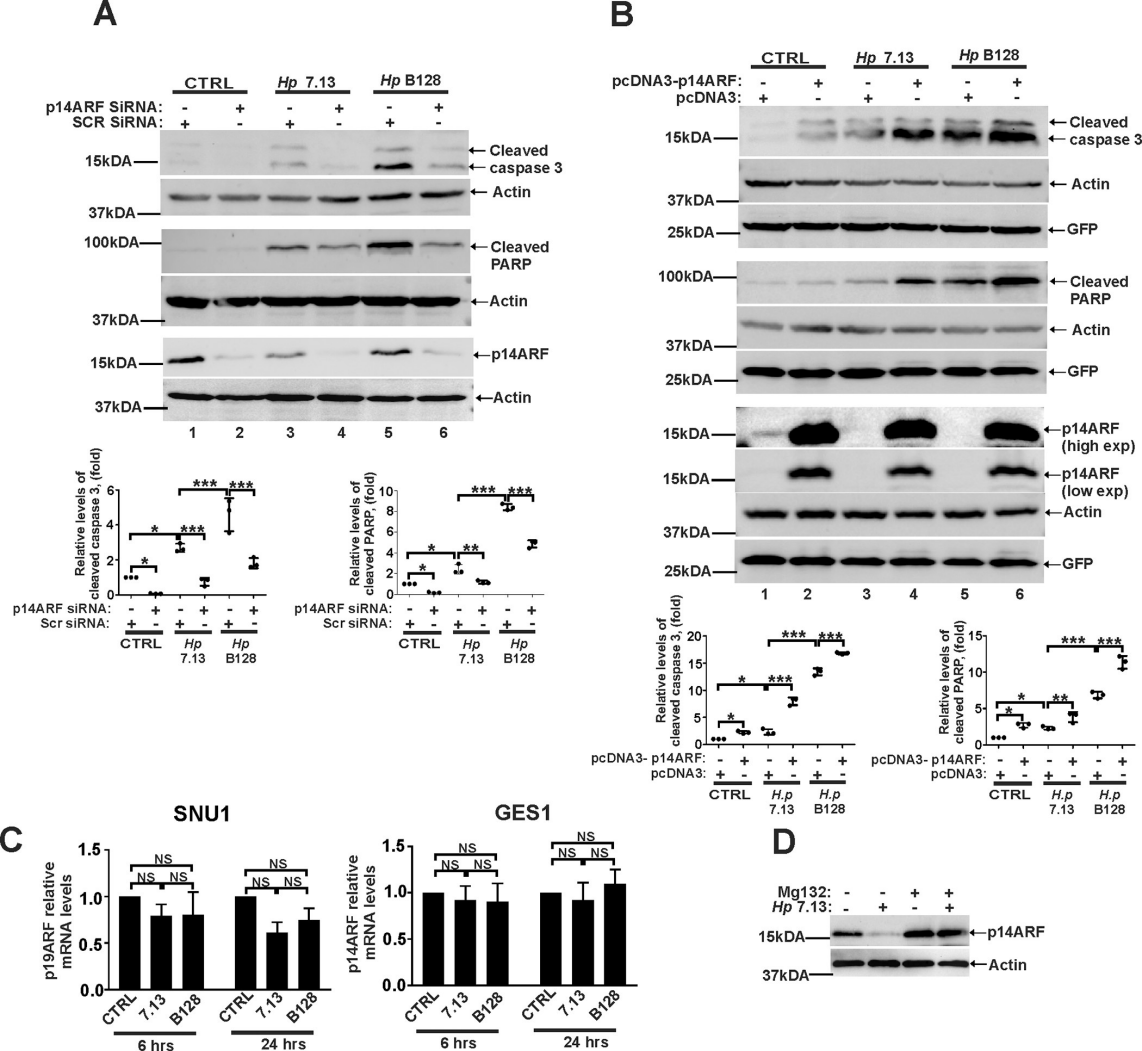
ARF regulates apoptosis in *H*. *pylori* infected cells. (A) Western blot analysis of apoptosis markers, cleaved caspase 3 and PARP in SNU1 cells transfected with p14ARF siRNA or control scrambled siRNA (Scr siRNA) and then either co-cultured with *H*. *pylori* strains 7.13 or B128 for 18 hours or left uninfected. Each experiment was carried out three times (n = 3), with representative blots displayed. Bottom panels show the densitometric analyses. Protein expression levels in control cells were arbitrarily set at 1. (B) The same as (A), but AGS cells were transfected with pcDNA3-p14ARF expression plasmid or empty pcDNA3 vector. (C) qPCR analysis of p14ARF mRNA in SNU1 and GES1 cells co-cultured with *H*. *pylori* strains 7.13 or B128 for the indicated time periods. Expression of p14ARF mRNA in uninfected cells was arbitrarily set at 1. (D) SNU1 cells co-cultured with *H*. *pylori* strain 7.13 were treated with proteasome inhibitor MG132 (20μM) or DMSO (vehicle), and analyzed for p14ARF protein expression by Western blotting (n = 3). Data in (A, B and C) were analyzed using 1-way ANOVA followed by Tukey’s multiple comparison test. Data are displayed as mean ± SD.

To assess the biological impact of ARF downregulation, we analyzed cleaved caspase 3 and PARP proteins that are known apoptosis markers. ARF was downregulated with specific siRNA in SNU1 cells that were then co-cultured with the indicated *H*. *pylori* strains. SNU1 was the gastric cell line of choice because it expresses high levels of endogenous ARF. We found that while *H*. *pylori* bacteria expectedly damage cells and increase apoptosis, downregulation of ARF significantly inhibits it (Fig 2A; compare lanes 3 and 5 with 4 and 6). In complementary experiments, we increased levels of ARF by transfecting AGS cells with p14ARF-expression plasmid (pcDNA3-p14ARF). Apoptosis was then analyzed as described above. In this series of experiments AGS cells were chosen due to their high transfectability that was normalized to GFP expression. Analyzing transfected AGS cells, we found that ARF increases apoptosis induced by *H*. *pylori*, further implicating ARF in regulation of cell death in *H*. *pylori* infected cells (Fig 2B). This conclusion was further confirmed using TUNEL assay (S2 Fig).

### E3 ubiquitin ligases SIVA1 and ULF regulate p14ARF in infected cells

Next, we explored how ARF is regulated in *H*. *pylori*-infected cells. Levels of p14ARF mRNA were measured by qPCR at the indicated time points after co-culture of SNU1 and GES1 with *H*. *pylori* strains 7.13 and B128. No significant changes in ARF mRNA levels (Fig 2C) were found, suggesting that ARF protein is regulated by posttranslational mechanisms. Actually, when protein expression of p14ARF was analyzed in the presence of proteasomal inhibitor MG132 (20 μM), this drug significantly inhibited downregulation of ARF caused by *H*. *pylori* (Fig 2D). Concentration of MG132 was selected based on previous studies showing no adverse effects on *H*. *pylori* activities [21,22].

**Fig 3:**
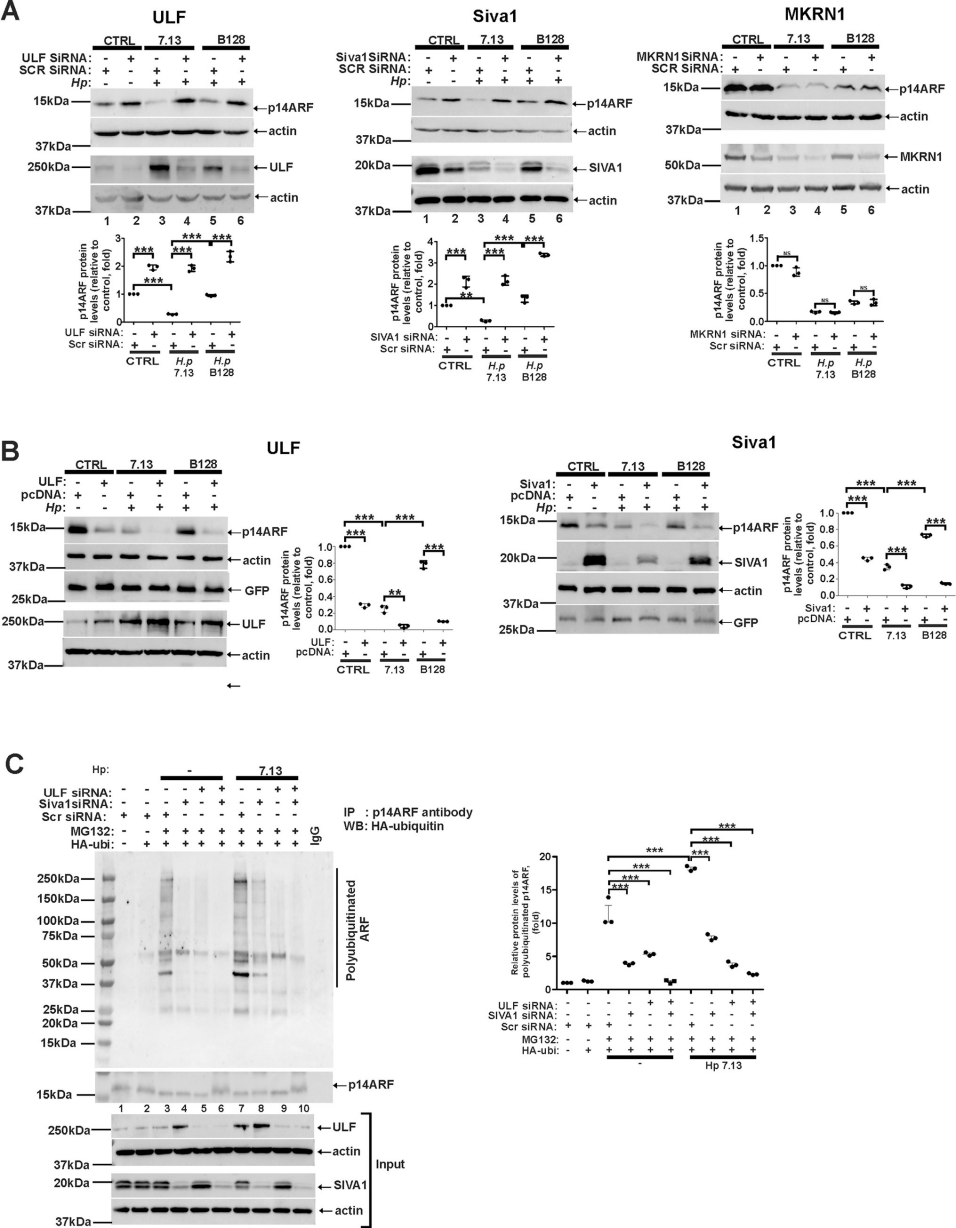
Downregulation of ULF and SIVA1 proteins inhibits ubiquitination and degradation of p14ARF protein in *H*. *pylori*-infected cells. **(**A) Analysis of p14ARF protein expression after downregulation of ULF (left panel), SIVA1 (central panel), or MKRN1 (right panel) with specific siRNAs in SNU1 cells co-cultured with *H*. *pylori* strains 7.13 or B128 for 4hrs or left uninfected. Transfections with scrambled siRNA (Scr siRNA) were used as controls (n = 3). (B) The same as (A), but SNU1 cells were transfected with plasmids expressing ULF (left panel) or SIVA1 (right panel) proteins. Transfections with empty vector (pcDNA3) were used as controls (n = 3). (C) Analysis of p14ARF protein ubiquitination after downregulation of ULF or SIVA1 with siRNAs in SNU1 cells co-cultured with *H*. *pylori* strain 7.13. Bottom panels show expression of ULF and SIVA1 in input cell lysates. All experiments were performed at least three times (n = 3) with representative blots shown. Graph panels depict the densitometric analyses. Protein expression levels in control cells were arbitrarily set at 1.

Given that several E3 ubiquitin ligases SIVA1, ULF, and MKRN1 [10–12] have been implicated in ubiquitination and degradation ARF protein, they were systematically downregulated with specific siRNAs in SNU1 cells that were then co-cultured with *H*. *pylori* strains 7.13 or B128 and analyzed for expression of p14ARF protein. Transfections with scrambled siRNA were used as controls. Our studies revealed that downregulation of ULF (Fig 3A; left panel) and to a lesser extent SIVA1 (Fig 3A; center panel), but not MKRN1 (Fig 3A; right panel) reproducibly inhibited downregulation of ARF protein. Notably, this effect was significantly more evident after co-culture of gastric cells with strain 7.13 than B128 (Fig 3A; compare lanes 3 and 4 with 5 and 6). This result is consistent with our observations shown above that strain 7.13 has a stronger effect on ARF than B128. To further validate our findings, expression of SIVA1 and ULF proteins were increased by transfections of ULF and SIVA1 expression plasmids. We found that elevated expression of ULF (Fig 3B; left panel) or SIVA1 (Fig 3B; right panel) result in decreased levels of p14ARF implicating them in regulation of ARF protein in infected cells.

To investigate whether SIVA1 and ULF affect p14ARF protein ubiquitination, SNU1 cells were co-transfected with vector expressing HA-tagged ubiquitin and either ULF siRNA or SIVA1 siRNAs or control scrambled siRNA, treated with MG132 proteasomal inhibitor (20 μM) and co-cultured with *H*. *pylori* strain 7.13 for 4 hours as this strain has a stronger effect on ARF. Ubiquitination of p14ARF protein was then assessed after its immunoprecipitation with ARF antibody. Immunoprecipitation with nonspecific IgGs was used as a control (Fig 3C). Our results showed that downregulation of ULF and SIVA1 reduces p14ARF polyubiquitination in infected cells with ULF siRNA having a significantly stronger effect (Fig 3C; compare lanes 8 and 9). Taking together, we concluded that ULF plays an important role in the regulation of ARF protein in *H*. *pylori*-infected cells. SIVA1, but not MKRN1, is also involved in this process.

**Fig 4:**
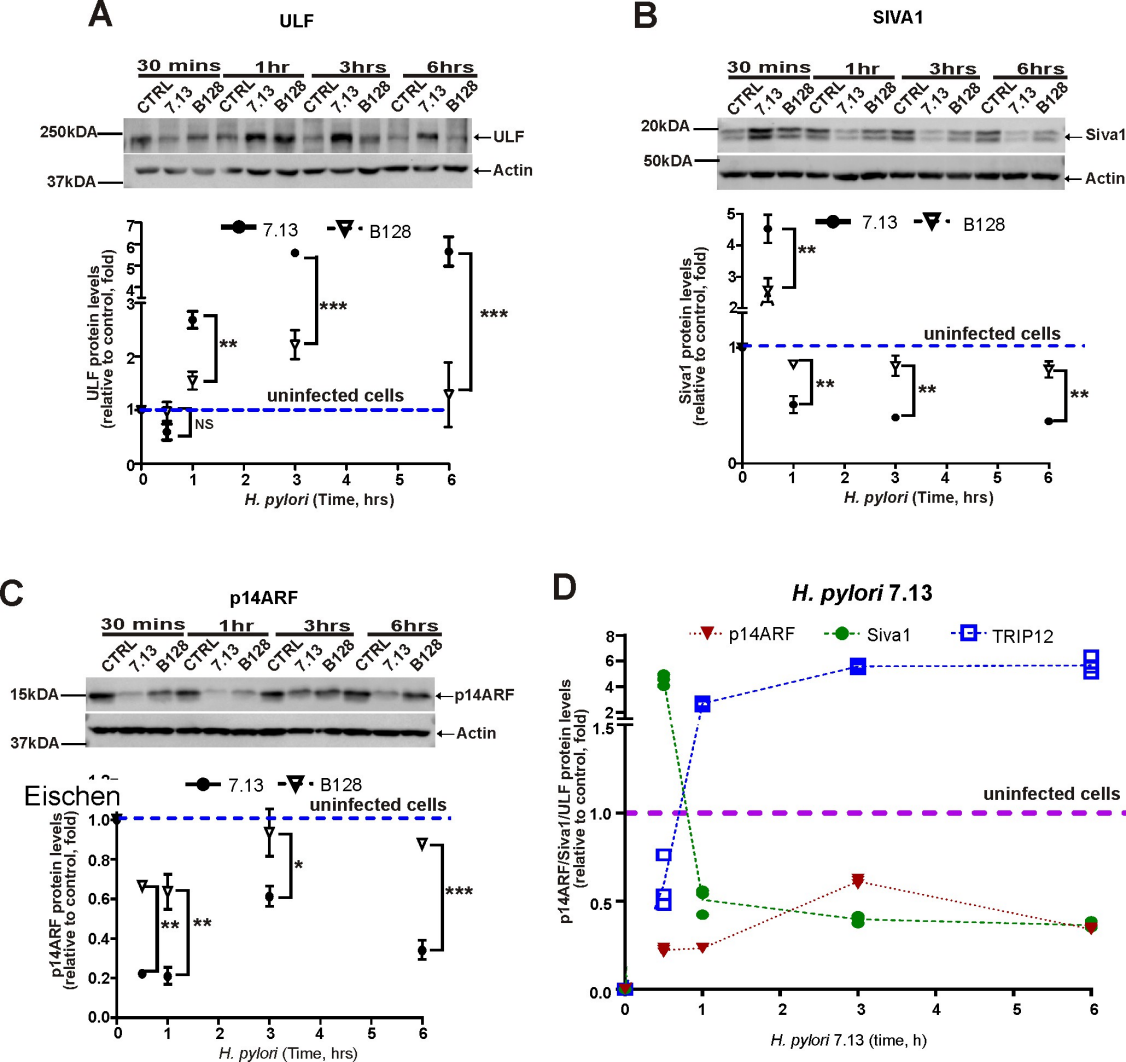
Protein levels of ULF, SIVA1, and p14ARF are dynamically changed in *H*. *pylori*-infected cells. Protein expression of ULF (A), SIVA1 (B) and p14ARF (C) in SNU1 cells co-cultured with strains 7.13 or B128 for the indicated time periods. Bottom panels show the densitometric analyses. (D) Temporal dynamics of ULF, SIVA1, and p14ARF proteins in SNU1 cells co-cultured with *H*. *pylori* strain 7.13. Statistical analysis was performed using 1-way ANOVA with Tukey’s multiple comparison test. Data are displayed as mean ± SD; n = 3.

Next, we assessed temporal changes in protein levels of ULF and SIVA1 after co-culture of SNU1 cells with *H*. *pylori* strains 7.13 or B128. Our analyses found that ULF protein levels are increased after infection in a time-dependent manner (Fig 4A; bottom panel shows the densitometric measurement of ULF protein levels; dashed line depicts the median levels of ULF protein in uninfected cells). Upregulation of ULF was followed by strong downregulation of ARF protein (Fig 4C). In contrast to ULF, SIVA1 levels rapidly downregulated with a short temporary increase at 30 min after *H*. *pylori* infection (Fig 4B). Combined dynamics of ULF, SIVA1 and ARF proteins are shown in Fig 4D. These protein changes were not accompanied by significant alterations in mRNA levels in analyzed cell lines (Figs 2C and S1B and S1C). Taken together, our studies suggest that *H*. *pylori* inhibits activity of ARF protein by affecting ARF ubiquitin ligases. Notably, tumorigenic strain 7.13 was significantly more potent in induction of ULF and downregulation of SIVA1 and ARF proteins (Figs 4 and S3A). We also concluded that whereas SIVA1 regulates ARF in uninfected cells and at initial stages of infection (plausibly until SIVA1 levels are sufficiently decreased), induction of ULF is primarily responsible for downregulation of ARF in *H*. *pylori*-infected cells.

### Regulation of SIVA1 and ULF proteins during *H*. *pylori* infection *in vivo*

**Fig 5:**
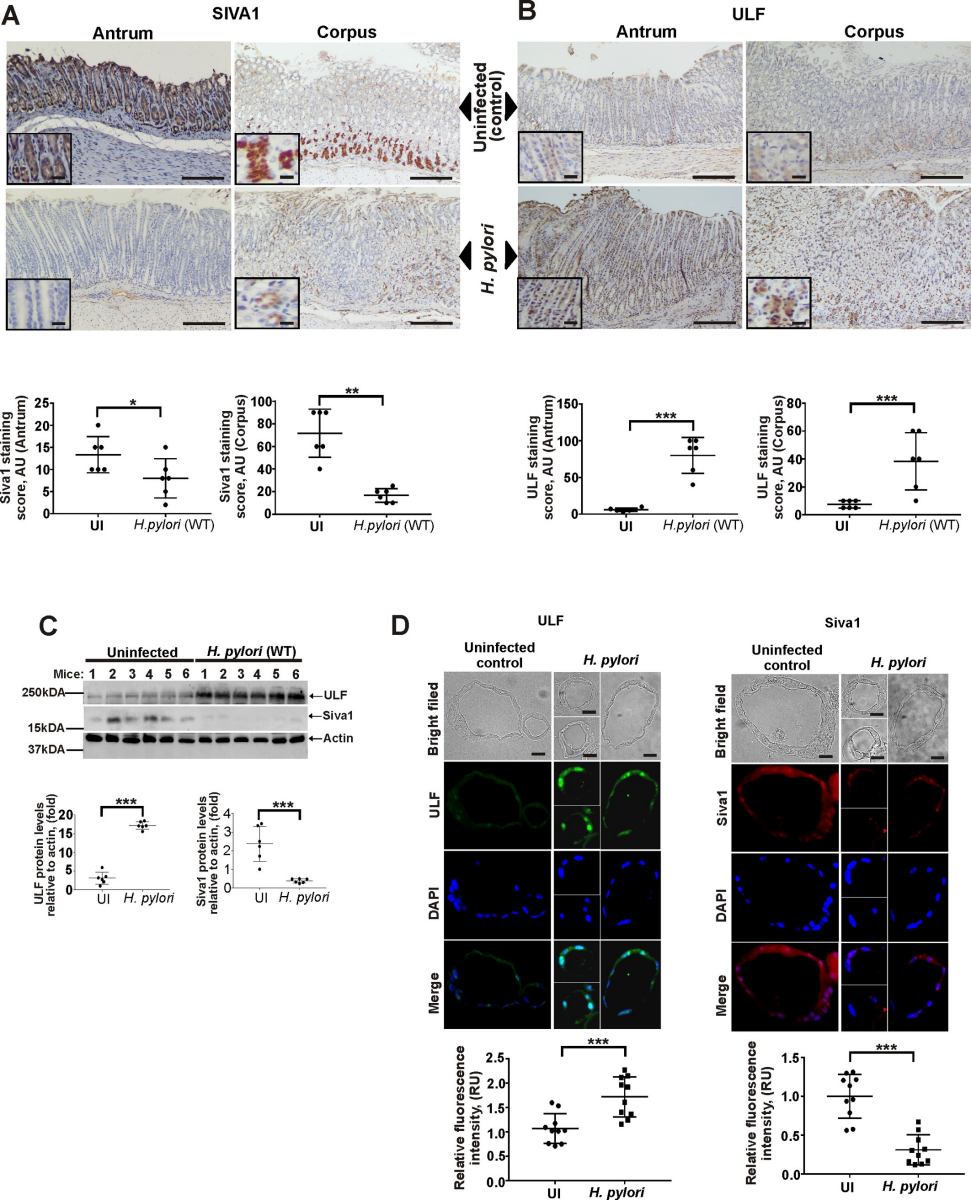
Analyses of ULF and SIVA1 protein expression in the murine stomach. (A) A representative image showing IHC staining for SIVA1 protein in the corpus and antrum of *H*. *pylori*-infected and non-infected mice (n = 6/group). (B) The same as (A) but ULF protein staining is shown. (C) The same as (A and B), but Western blotting was used for analyses of ULF and SIVA1 proteins in gastric tissue homogenates. Bottom panel shows the densitometric analyses. (D) Protein expression of ULF (left panel) and SIVA1 (right panel) in gastric organoids derived from the antropyloric region of the murine stomach that were infected with *H*. *pylori* strain 7.13 *in vitro* or left uninfected. Panels show representative light microscopic and immunofluorescence images. Bottom panel shows quantification of ULF and SIVA1 fluorescence intensity/cell. Data were analyzed using unpaired 2-tailed t-test. Data are displayed as mean ± SD.

We next investigated how ULF and SIVA1 proteins are regulated in *H*. *pylori*-infected animals. Expression of SIVA1 and ULF proteins were assessed in the murine stomach after infection with *H*. *pylori* strain PMSS1 for 10 days using IHC and Western blotting. In uninfected animals, SIVA1 protein was primarily expressed in chief cells toward the base of the oxyntic glands, antral foveolar cells, and at the base of the gastric antral glands (Fig 5A). Only low levels of ULF were detected in epithelial cells in the same tissues (Fig 5B). In contrast, *H*. *pylori* infection led to strong upregulation of ULF protein and downregulation of SIVA1 in the stomach (Fig 5A and 5B). These results were consistent with changes found in gastric homogenates analyzed by Western blotting (Fig 5C). We were also able to recapitulate effects of *H*. *pylori* on ULF and SIVA1 in the murine gastroids derived from the antropyloric region after their infection with *H*. *pylori* strains 7.13 or PMSS1 *in vitro* (Figs 5D and S3B, respectively).

To analyze the consequences of inhibition of ARF, we conducted long-term experiments, where we compared p19ARF null mice, which have homozygous deletion of the INK4A/ARF locus (*arf*^*-/-*^), and control wild type animals infected with *H*. *pylori* strain PMSS1 for one year or longer (n = 10, per group). We found that *H*. *pylori* infection of ARF null mice leads to chronic inflammation in the stomach and 6/10 (60%) of animals developed gastric dysplasia and tumors at 8–10 months after initial infection (S4B and S4C Fig). No gastric tumors were found in infected wild type mice or control uninfected ARF null animals, implicating ARF in inhibition of gastric carcinogenesis.

**Fig 6:**
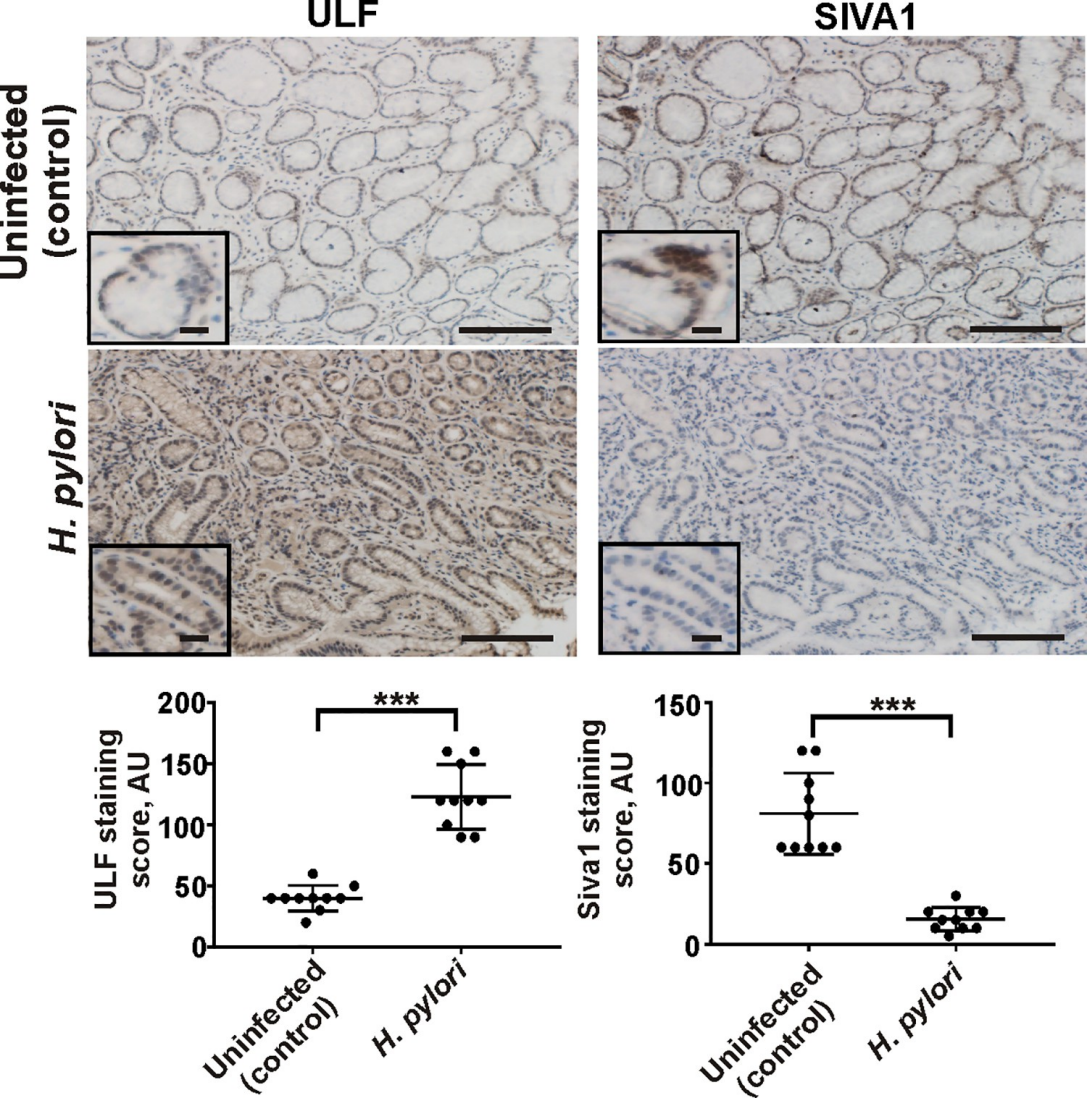
Infection with *H*. *pylori* alters expression of ULF and SIVA1 proteins in the human stomach. Representative images showing IHC staining for ULF and SIVA1 proteins in antral gastric biopsies collected from *H*. *pylori*-infected and non-infected patients with active chronic gastritis. The graph panels show the IHC scores (n = 10/group). Data were analyzed using unpaired 2-tailed T-test. Insets show the magnified views (×40). Scale bars: 50 μm.

We next analyzed SIVA1 and ULF proteins in gastric antral biopsies collected from *H*. *pylori*-infected and uninfected patients (n = 10/group). Similar to analyses of infected murine gastric tissues, protein levels of ULF were found to be significantly increased in *H*. *pylori*-infected subjects compared to ones without infection (Fig 6; left panels). Increase in protein levels of ULF was accompanied by downregulation of SIVA1 (Fig 6; right panels). Combined, our data demonstrate that *H*. *pylori* alters expression of E3 ubiquitin ligases that regulate p14ARF in the human and murine stomachs in vivo.

### Bacterial C*agA* protein regulates SIVA1 and ULF E3 ubiquitin ligases and targets p14ARF protein to degradation

**Fig 7:**
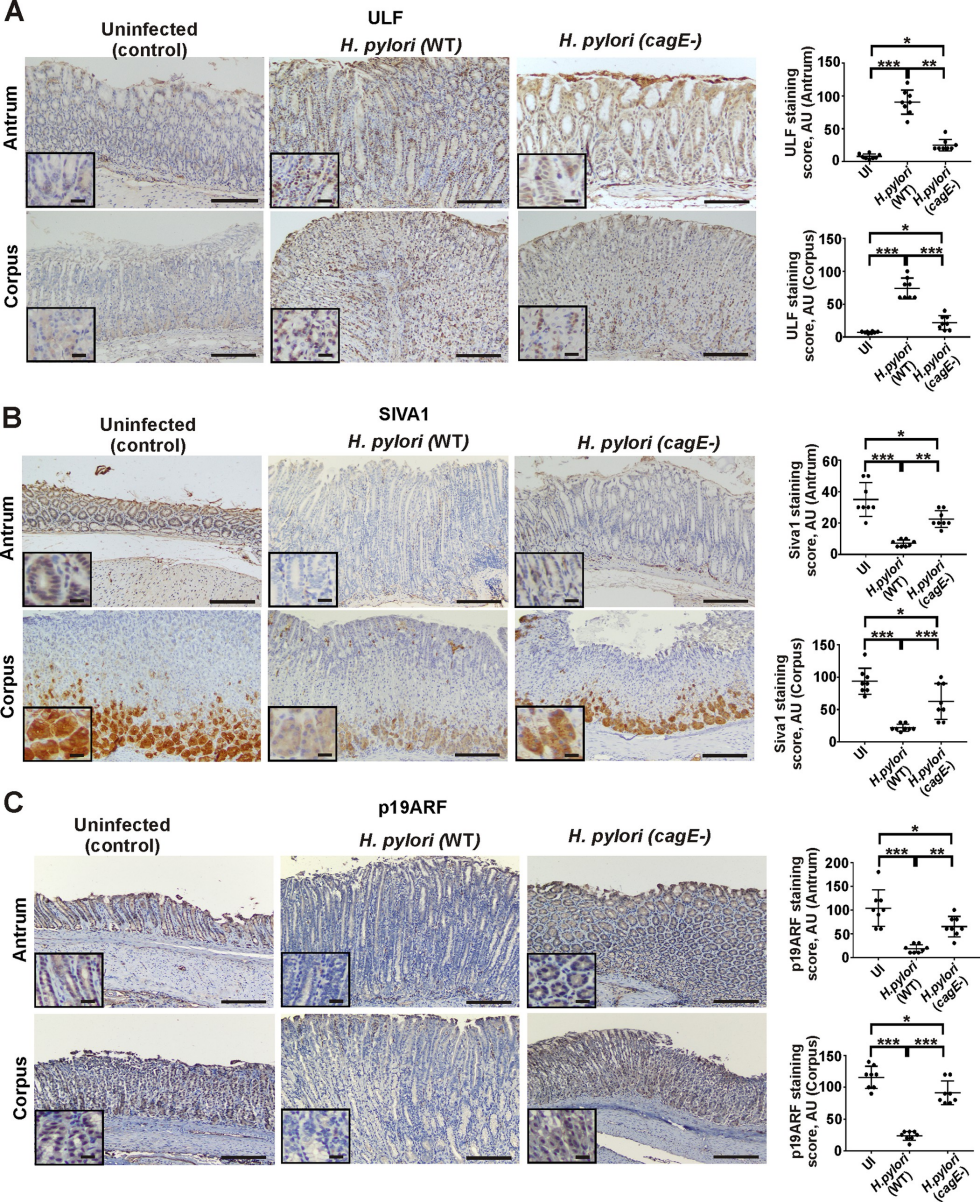
The T4SS affects ULF, SIVA1, and p19ARF proteins in the murine stomach. **(**A) Representative IHC images showing expression of ULF (A), SIVA1 (B), and p19ARF (C) proteins in the corpus and the antrum of mice infected with wild type *H*. *pylori* strain PMMS1 or its *cagE-* isogenic mutant for 8 weeks (n = 6/group). Uninfected mice were used as controls. The graph panels show the corresponding IHC scores. Statistical analysis was performed using 1-way ANOVA with Tukey’s multiple comparison test. Data are displayed as mean ± SD. Insets show the magnified views (×40). Scale bars: 50 μm.

Given the important role of the T4SS in *H*. *pylori* pathogenesis, we analyzed mice infected with *H*. *pylori* PMSS1 *cagE- isogenic* mutant, which has defective T4SS and is unable to deliver CagA into host cells [23]. We found that whereas infection with wild type strain PMSS1 leads to significant increase of ULF levels (Figs 7A and S4A) and downregulation of SIVA1 (Figs 7B and S4A), as well as p19ARF (Figs 7C and S4A) in the corpus and antrum, *cagE-* mutant has a diminished ability to do so, suggesting an important role of the T4SS signaling.

**Fig 8:**
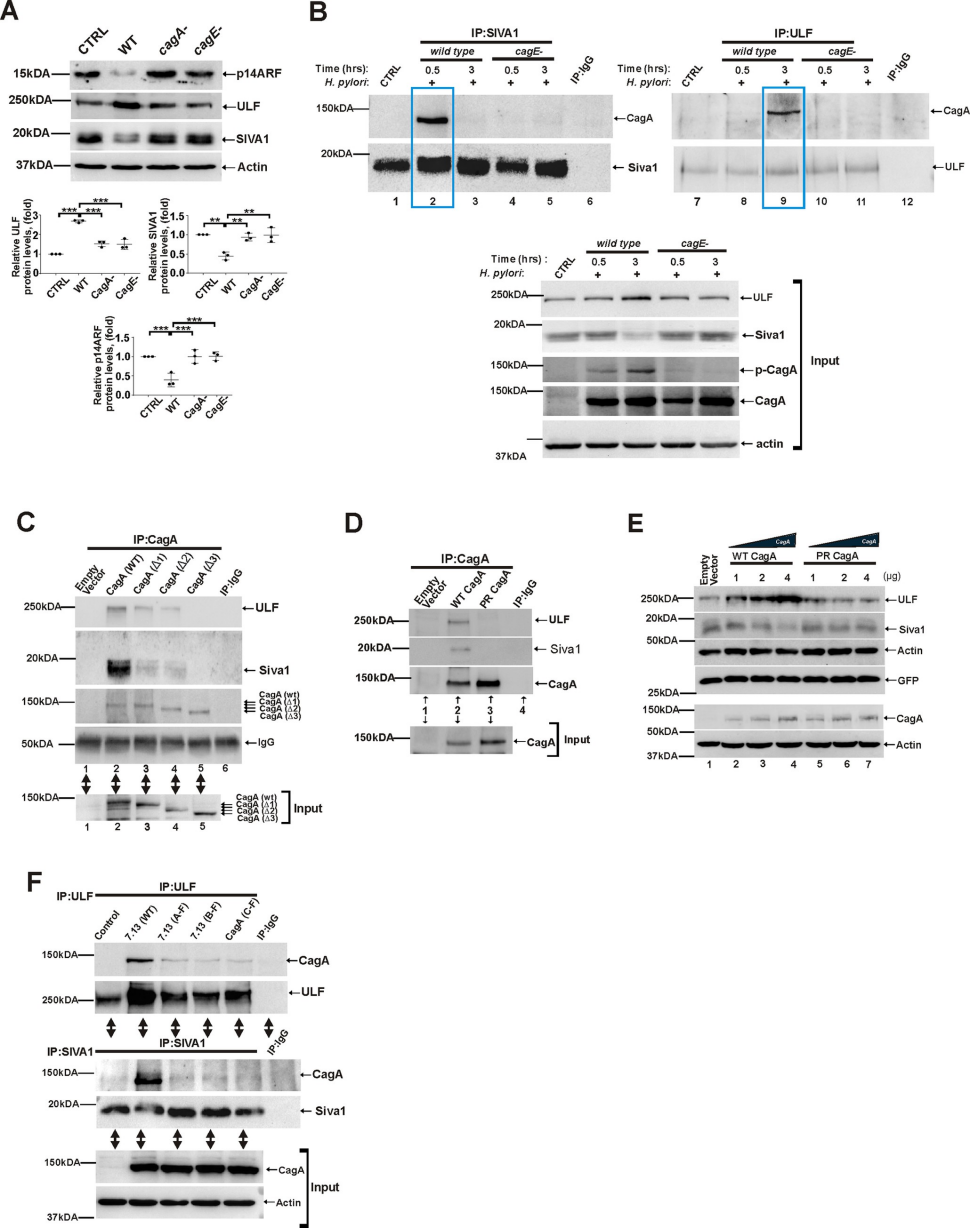
Bacterial CagA protein interacts with ULF and SIVA1. (A) Expression of ULF, SIVA1 and p14ARF proteins in SNU1 cells co-cultured with *H*. *pylori* strain 7.13 or its *cagA*- and *cagE*- isogenic mutants for 6 hrs. Bottom panels show the densitometric analyses of ULF, SIVA1, and p14ARF proteins. (B) Co-immunoprecipitation analysis of CagA binding to ULF and SIVA1 proteins in SNU1 cells co-cultured with *H*. *pylori* strain 7.13 and its isogenic *cagE-* mutant for the indicated time periods. Equal amounts of total cell lysates were immunoprecipitated using ULF and SIVA1 antibodies and analyzed for CagA binding using anti-CagA antibody (n = 3). Immunoprecipitations with non-specific mouse and rabbit antibodies were used as controls. Two boxed areas show binding of CagA to ULF and SIVA1 proteins after their immunoprecipitation. (C) Co-immunoprecipitation analysis of CagA binding to ULF and SIVA1 proteins in AGS cells that were transfected with plasmids expressing wild type CagA or ΔEPIYA-CagA deletion mutants. Equal amounts of total cell lysates were immunoprecipitated using CagA antibody and analyzed for ULF and SIVA1 binding to CagA. Bottom panel shows input. (D) The same as (C), but AGS cells were transfected with wild type CagA expression plasmid or phosphorylation-deficient CagA mutant (PR CagA). (E) Regulation of ULF, SIVA1, and p14ARF proteins in SNU1 cells transfected with an increasing amount of wild type CagA- and PR CagA- mutant plasmids. Co-transfection with GFP-expression plasmid was used to normalize for differences in transfection efficiency. (F) Assessment of the role of the EPIYA motifs. SNU1 cells were co-cultured with wild type *H. pylori* strain 7.13 or its phosphorylation-deficient mutants 7. 13 (A-F), 7. 13 (B-F), or 7. 13 (C-F) and analyzed for binding of CagA to Siva1 and ULF at various time points. Interactions of SIVA1 and ULF proteins with CagA are shown at 0.5hrs and 3hrs, respectively. Bottom panel shows input. All experiments were performed three times (n = 3) with representative blots shown.

To assess roles of the T4SS and CagA, SNU1 cells were co-cultured with wild type *H*. *pylori* strain 7.13 and its *cagA-* (7.13 *cagA-*) and *cagE-* (7.13 *cagE-*) isogenic mutants and analyzed for expression of endogenous ULF, SIVA1 and p14ARF proteins (Fig 8A). The latter strain was used as an additional control since it is able to produce CagA, but has the defective T4SS. This was in an agreement with our analyses of CagA tyrosine phosphorylation showing that only wild type bacteria deliver CagA into host cells that becomes phosphorylated (S5A Fig). Our analyses also showed that while parental wild type bacteria strongly upregulate ULF and downregulate SIVA1 and ARF, both *cagA-* or *cagE-* mutants are defective in these functions, implicating cagA in regulation of ULF and SIVA1 (Fig 8A).

Since CagA often affects host proteins through protein-protein interactions [2], we next assessed whether CagA physically interacts with SIVA1 and ULF in cells infected with wild type *H*. *pylori* strain 7.13 or its *cagE-* isogenic mutant. CagA protein tyrosine phosphorylation and its expression are shown in Fig 8B (input, bottom panels). Following co-culture with *H*. *pylori*, endogenous SIVA1 and ULF proteins were immunoprecipitated with specific antibodies and analyzed for CagA binding at various time points. Surprisingly, we found that both ULF and SIVA1 proteins bind to CagA protein in a sequential manner. Strong binging of CagA to SIVA1 was observed at early time points (approx. 30 min after infection) as shown in Fig 8B (compare lane 2 with lanes 1 and 3). Analyses of infected cells collected at later time points did not find significant binding of SIVA1 to CagA, but showed strong ULF-to-CagA interactions (Fig 8B; compare lane 9 with lanes 7 and 8) demonstrating that binding of CagA with SIVA1 and ULF is sequential. No interactions between SIVA1/ULF and CagA were found in cells co-cultured with *cagE-* mutant further confirming our findings (Fig 8B).

To exclude potential effects of other bacterial factors and, AGS cells were transfected with CagA-expressing plasmid and analyzed for SIVA1 and ULF binding using co-immunoprecipitation assay. As an additional control, interaction of CagA with MKRN1 was also assessed. Our analyses showed that SIVA1 and ULF proteins (but not MKRN1) strongly interact with CagA (Figs 8C (lane 2) and S5B).

To better understand the nature of these interactions, we generated SIVA1 and ULF deletion mutants and analyzed their binding to CagA (deletion scheme for SIVA1 and ULF is shown in S5C Fig). Our data showed that the WWE domain of ULF and the C- and N- termini of SIVA1 are involved in these interactions (S5C Fig).

We next assessed whether phosphorylation of CagA protein plays a role in the CagA binding. Given that CagA is phosphorylated at the EPIYA motifs, we constructed CagA deletion mutants, which lack one(Δ1), two(Δ2) or three(Δ3) EPIYA motives, and analyzed for their binding to ULF and SIVA1. We found that removing even one of the EPIYA motifs significantly decreases the binding of CagA to SIVA1 and ULF and Δ3 mutant loses its binding ability (Fig 8C; compare lanes 2 with 3, 4 and 5)).

To further corroborate our findings, we took advantage of CagA phosphorylation-deficient mutant (PR CagA), in which tyrosine residues within the EPIYA motifs are replaced with alanine [24]. We found that only wild type CagA, but not PR CagA mutant, binds to ULF and SIVA1 proteins (Fig 8D). Moreover, when cells were transfected with an increasing amount of wild-type CagA or PR CagA mutant, only wild type CagA, but not PR CagA, induces degradation of SIVA1 and upregulation of ULF proteins in a dose dependent manner (Fig 8E).

Next, we generated *H*. *pylori* phosphorylation mutants that express cagA with amino acid substitutions (Tyr to Phe) within the A, B, or C EPIYA motives. Cells were then co-cultured with these mutants and analyzed for interactions of CagA with SIVA1 and ULF proteins at various time points. Similar to experiments shown above, phosphorylation-deficient mutants show a diminished ability to interact with ULF and SIVA1 proteins (Fig 8F). Taken together, our data show that CagA interacts with host proteins ULF and SIVA1 in a phosphorylation-specific manner.

## Discussion

Our study provides the first evidence that *H*. *pylori* CagA protein reorganizes the ubiquitin-proteasome system that controls protein levels of p14ARF, the key regulator of the oncogenic stress response. We reproducibly observed upregulation of ULF and downregulation of SIVA1 and ARF proteins in the gastric mucosa of *H*. *pylori*-infected mice and human individuals, as well as were able to recapitulate these changes *in vitro* using gastroids and gastric cell cultures. Our analyses of *H*. *pylori* isogenic mutants signified importance of the T4SS. We found that *H*. *pylori cagA-* and *cagE*- mutants, which are deficient in CagA delivery, have diminished ability to regulate SIVA1, ULF, and p14ARF. CagA was found to reproducibly bind to SIVA1 and ULF E3 ubiquitin ligases in a sequential fashion, potentially functioning as a ubiquitin ligase switch that regulates p14ARF during *H*. *pylori* infection (Fig 8). We found that interactions of CagA with ULF and SIVA1 are dependent on tyrosine phosphorylation of CagA protein at the EPIYA motifs. The nature of these interactions is not completely clear given the lack of the known pTyr recognition domains (e. g. SH2) in Siva1 and ULF proteins. It is plausible that in this case, recognition of the CagA phoshotyrosines may be mediated by auxiliary cellular proteins. It is also possible that other domains may play a role. Indeed, our data show that WWE domain of ULF protein, which mediates specific protein-protein interactions in ubiquitin and ADP-ribose conjugation systems, is involved in CagA interactions. Additional studies are needed to further dissect these mechanisms.

It has been suggested that CagA functions as a scaffold protein that rearranges cell signaling pathways and alters important cellular processes [2]. Our data showed that the presence of CagA allows *H*. *pylori* bacteria to reshape the protein degradation machinery to inhibits ARF, which is involved in induction of apoptosis. Our studies demonstrated that downregulation of ARF diminishes apoptosis in *H*. *pylori*-infected cells. This may provide certain advantages to *H*. *pylori* bacteria e.g. suppression of host immune responses caused by apoptotic signaling [25]. However, it may also have a tumor-promoting effect as GC and many other human tumors are characterized by upregulation of ULF and inhibition of p14ARF [26]. This concept is in agreement with our findings showing that ARF knockout mice infected with *H*. *pylori* develop gastric cancerous lesions.

Importantly, *H*. *pylori* regulates E3 ubiquitin ligases ULF and SIVA1 in a strain specific manner. We found significant differences between *H*. *pylori* strain B128 and its carcinogenic derivative strain 7.13, which induce gastric cancer in various animal models [19,20]. Strain 7.13 was significantly more potent in upregulation of ULF and inhibition of SIVA1 and ARF. The latter can be plausibly explained by differences in CagA delivery. Although both strains 7.13 and B128 are *cagA*-positive, it has been demonstrated that *H*. *pylori* strain 7.13 translocates significantly larger amount of CagA protein into host cells than strain B128 [7,19]. In good agreement, our experiments show that CagA regulates SIVA1 and ULF in a dose dependent manner (Fig 8E). In overall, our findings are consistent with previous observations showing that quantitative and qualitative characteristics of bacterial and viral proteins and their variability play a pivotal role in the regulation of p53 and ARF [2,27].

One of the most important functions of ARF is regulation of the OSR that is responsible for inhibition of cell proliferation caused by aberrant induction of various oncogenes. *H*. *pylori* is known to activate Ras/ERK, WNT/β-catenin, PI3K/AKT and other oncogenic pathways [2]. When activated, ARF can trigger p53-dependent apoptosis. It can also induce apoptosis and other antiproliferative responses independently of p53 [28]. Our studies demonstrate that *H*. *pylori* downregulates p14ARF protein primarily by inducing ULF. SIVA1 may also contribute to ARF inhibition during initial stages of *H*. *pylori* infection until its level is significantly decreased. Downregulation of SIVA1 may provide additional benefits to bacteria as SIVA1 plays a proapoptotic role and its inhibition leads to suppression of apoptosis [29]. Additional studies are needed to fully explore the biological consequences of the interplay between ULF and SIVA1 in the settings of different *H*. *pylori* strains.

**Fig 9:**
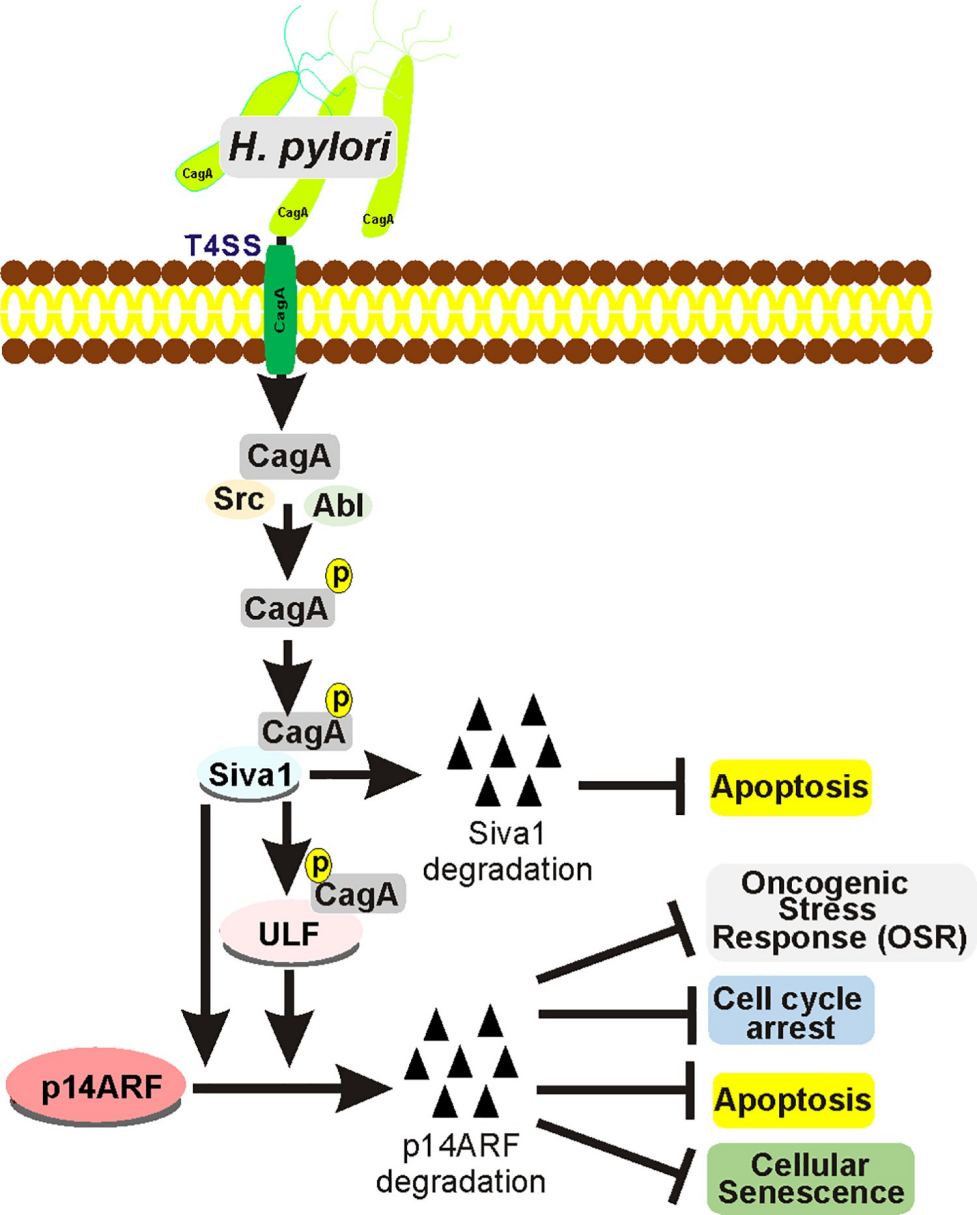
Scheme depicting the regulation of ULF, SIVA1 and p14ARF proteins during *H*. *pylori* infection.

In summary, our studies demonstrate that *H*. *pylori* exploits the ubiquitin-proteasome system to inhibit expression of p14ARF tumor suppressor affecting the host E3 protein ligases SIVA1 and ULF in a CagA-dependent manner (Fig 9). These findings may help to better understand the mechanisms of *H*. *pylori*-driven tissue damage and pathogenesis.

## Materials and methods

### Ethic statement

All human tissue specimens collected had personal identifiers removed. The study protocol was exempted from review by the Institutional Review Board of the University of Miami.

### Cell lines, *H*. *pylori* strains, and qPCR analysis

Human gastric epithelial cell lines AGS and SNU1 were purchased from ATCC (Manassas, VA). Human immortalized gastric epithelial cell line GES1 was obtained from Dr. El-Rifai (University of Miami) and described previously [30]. Cells were grown in Ham’s F12 (AGS, SNU1) or RPMI (GES1) media, supplemented with 10% Fetal Bovine Serum (Thermo Fisher Scientific, Waltham, MA), at *37°C* in a humidified atmosphere containing 5*%* CO_2_.

*H*. *pylori* strains B128, 7.13, and PMSS1, as well as their *cagA*- and *cagE*- isogenic mutants were described previously [19,20,31,32]. *H*. *pylori* strains with mutations in the *cagA* gene were received from Dr. Peek. These mutants were derived from parental *H*. *pylori* strain 7.13 by replacing tyrosine (Y) to phenylalanine (F) in the EPIYA motifs (A, B or C). Methods of *H*. *pylori* mutagenesis and complementation were previously reported [33].

Bacteria were cultured on Trypticase Soy Agar plates with 5% sheep blood (BD Biosciences, San Jose, CA) or in Brucella broth (BD Biosciences, San Jose, CA) containing 10% FBS and 10μg/ml vancomycin at 37°C in a humidified atmosphere in the presence of 5*%* CO_2_. Bacteria were harvested by centrifugation, resuspended in PBS and added to gastric cells at a bacteria-to-cell ratio of 100:1 (MOI 100).

Gastric cells were co-cultured with *H*. *pylori* and harvested in RIPA buffer containing protease and phosphatase inhibitors. Cell lysates were analyzed with Western blotting. Densitometry was performed using the ImageJ software (NIH, Bethesda). Cellular RNA was extracted using the Qiagen RNeasy Kit (Valencia, CA) and reverse transcribed with the Applied Biosystems High Capacity cDNA Reverse Transcription Kit (Carlsbad, CA) according to the manufacturers’ protocols. mRNA expression of p14ARF, SIVA1 and ULF were assessed by qPCR using the following primers:

p14ARF-F: 5’-TTTCGTGGTTCACATCCCGC-3’;

p14ARF-R: 5’-GGGCGCTGCCCATCATCA-3’;

SIVA1-F: 5′- TCTTCGAGAAGACCAAGCG −3’;

SIVA1-R: 5′- TGCCCAAGGCTCCTGATC −3’;

ULF-F:5’-TCAATGAGGACACGGGAACAG-3’;

ULF-R: 5’-GGCACTTATGTCTGACCGCA-3’;

HPRT-F-5’-TTGGAAAGGGTGTTTATTCCTCA-3’;

HPRT-R-5’-TCCAGCAGGTCAGCAAAGAA.

Expression of HPRT mRNA (Hypoxanthine-Guanine Phosphoribosyl Transferase) was employed as an endogenous control.

### Antibody, vectors, RNAi, apoptosis analysis, and transfections

The following antibodies were used for Western blotting and immunohistochemical staining: SIVA1 (sc-514375, sc-376260), GFP (sc-9996), and normal mouse IgG (sc-2025) from Santa Cruz Biotechnology (Santa Cruz, CA); p14ARF (ab185620) and p19ARF (ab80) from Abcam (Cambridge, MA); ULF/TRIP12 (A301-814A) and MKRN1 (A300-990A) from Bethyl Laboratories (Montgomery, TX); β-Actin (A5441) from Sigma-Aldrich (St. Louis, MO); HA.11 (#901501) from BioLegend (San Diego, CA); p-Tyr (05-321X) from Millipore; CagA (HPP-5003-9) from Austral Biologicals (San Ramon, CA); HRP-conjugated anti-mouse IgG (W4028) from Promega; cleaved PARP (#9541), cleaved caspase 3 (#9661), caspase 3 (#9662), normal rabbit IgG (#2729) and HRP-conjugated anti-rabbit IgG (#7074) from Cell Signaling Technology (Danvers, MA).

Plasmids expressing HA-ubiquitin, p14ARF, wild type CagA (WT-CagA) and its phosphorylation resistant mutant PR CagA were previously described. [32, 34]. SIVA1 expression plasmid pcDNA3.1-FSIVA1 was purchased from GenScript (OHu18390D, Piscataway, NJ). ΔEPIYA-CagA mutants were generated by deletion of the corresponding EPIYA motifs from WT-CagA: (Δ1), (Δ2), and (Δ3). ULF/TRIP12-expressing vector pAC-FTRIP12 was generous gift from Dr. Wei Gu. [35] GFP expression vector (EGFP-N1) was from Clontech Laboratories. Deletion mutants of ULF and SIVA1 were generated by PCR, using vectors pcDNA3.1-FSIVA1 and pAC-FTRIP12 as templates, and confirmed by sequencing. The schema of deletions is shown in S5C Fig.

siRNAs against SIVA1 (5′-CACAGCACUUUCUCGUACAUGUCACUG-3’), p14ARF (5′-CAUGGUGCGCAGGUUCUUGGUGACC-3′), ULF (5′-CCAGGAGCAA-CAACUGAAAUCUGCA-3′) and MKRN1 (5’-GGUGUGGUUGCAGUUGGAGAGGAUCCC-3’) were synthesized by Integrated DNA Technologies (Coralville, Iowa). Scrambled control siRNA was purchased from Ambion (Grand Island, NY).

Transfections of plasmids and siRNAs were performed with Lipofectamine 2000 (Thermo Fisher Scientific, Waltham, MA) according to the manufacturer’s protocol. Proteasomal inhibitor MG132 was purchased from EMD Millipore (Burlington, MA). Cellular apoptosis was measured by analyzing cleaved PARP and caspase 3 proteins [17], or by TUNEL assay using *In Situ* Cell Death Detection Kit from Sigma-Aldrich (St. Louis, MO).

### Animal studies

Animal studies were performed according to the protocol approved by the Institutional Animal Care Committee of the University of Miami. Infection of mice with *H*. *pylori* was described previously [17,32]. Briefly, 4- to 8-week-old C57BL/6 mice were randomly assigned into control and experimental groups and orally gavaged with either sterile Brucella broth or rodent-adapted *H*. *pylori* strain PMSS1 or its isogenic mutant *cagE-*. Gastric tissues were collected 10 days or 8 weeks post-infection.

C57BL/6 mice with homozygous deletion of the INK4A/p19ARF locus was previously described [36]. To study long-term effects of *H*. *pylori* infection, p19ARF null and control wild type mice were orally gavaged with *H*. *pylori* strain PMSS1. Gastric tissues were collected 8–12 months post-infection. Collected tissues were fixed in 10% neutral-buffered formalin, paraffin-embedded, and analyzed by immunohistochemistry. Gastric tissues were also homogenized in RIPA buffer and analyzed by Western blotting.

Gastroid cultures were described previously [37]. Briefly, the gastric glands were isolated from 8- to 12-week-old C57BL/6 mice and co-cultured with *H*. *pylori* strains 7.13 or PMSS1 in advanced DMEM/F12 medium without penicillin/streptomycin for 2h. The infected and control uninfected gastric glands were embedded into Matrigel (BD Biosciences) and overlaid with the gastroid growth medium for ten days. Media were replaced every 3 days. Gastroid cultures were fixed in 10% neutral-buffered formalin for 30 min, washed in PBS, and resuspended in 30  μl of the HistoGel (Richard-Allan). The solidified specimens were placed into cassettes and processed for immunofluorescence analyses using antibodies against SIVA1 (Santa Cruz Biotechnology; sc-376260) and ULF/TRIP12 (Thermo Fisher Scientific; PA5-57840) at a 1:200 dilution. Secondary antibodies were purchased from Life Technologies (A11004) and Abcam (ab150071). Protein expression (immunofluorescence/per cell) was assessed using the ImageJ software.

### Human gastric tissue specimens

Antral gastric biopsies were collected from patients with active chronic gastritis and dyspeptic symptoms. Expression of SIVA1, ULF and p14ARF proteins were analyzed by IHC in gastric biopsies collected from individuals with or without H. pylori infection. Immunohistochemical staining was evaluated for staining frequency and intensity, as previously described [7]. The infection status was determined by a scope test.

### Analysis of p14ARF protein ubiquitination, protein immunoprecipitations

SNU1 cells were co-transfected with HA-ubiquitin-expressing plasmid, SIVA1 siRNA or/and ULF siRNA. Twenty-four hours after transfection, cells were treated with proteasomal inhibitor MG132 (20 μM) for 1 hour and co-cultured with *H*. *pylori* strain 7.13 for an additional 4 hrs. Cells were then lysed in RIPA buffer at 4°C. p14ARF protein was immunoprecipitated and analyzed for protein ubiquitination by Western blotting using anti-HA-tag or p14ARF antibodies. Immunoprecipitations with the corresponding non-specific IgGs were used as controls. Since AGS cells express low levels of endogenous p14ARF protein, they were transfected with p14ARF-expressing vector prior analysis of p14ARF protein ubiquitination.

Co-immunoprecipitation assays were performed to analyze interactions between bacterial virulence factor CagA and host proteins SIVA1 and ULF. Proteins were immunoprecipitated from cellular extracts with the corresponding antibodies and protein A/G agarose or Anti-FLAG affinity beads (Sigma-Aldrich, St. Louis, MO).

### Statistical analysis

GraphPad Prism 7 was used for statistical analysis. Depending on the data set, one-way ANOVA followed by Tukey’s multiple comparison test or two-tailed Student’s t-test were used. Results were presented as mean ± SD. Results were considered significant if p < 0.05 (*p < .05, **p < .01 ***p < .001, ns p > .05).

## Supporting information

S1 Fig Analyses of ULF and SIVA1 mRNA. (A) Representative images showing IHC staining of p14ARF protein in the human stomachs of uninfected and *H*. *pylori*-infected subjects. (B) qPCR analysis of ULF and SIVA1 mRNA in SNU1 cells co-cultured with *H*. *pylori* strains 7.13 or B128 for the indicated time. (C) The same as (B) but GES1 cells were analyzed.
(TIF)

S2 Fig p14ARF protein is involved in apoptosis induced by *H. pylori*. (A) TUNEL analysis was performed in SNU1 cells transfected with p14ARF siRNA or control scrambled siRNA and then either co-cultured with *H*. *pylori* strains 7.13 or B128 for 18 hours or left uninfected. (B) The same as (A), but AGS cells were transfected with pcDNA3-p14ARF expression plasmid or empty pcDNA3 vector. Each experiment was carried out three times (n = 3). Bottom panels show quantification of TUNEL staining. Data were analyzed using one-way ANOVA. Data are displayed as mean ± SD.
(TIF)

S3 Fig (A) Expression analysis of ULF and SIVA1 proteins in GES1 cells. GES1 cells were co-cultured with *H. pylori* strains 7.13 or B128 for 6 hours and analyzed for expression of ULF and SIVA1 proteins by Western blotting. (B) Analyses of expression of SIVA1 and ULF proteins in gastric organoids derived from the antropyloric region of the murine stomach that were infected with *H. pylori* strain PMSS1 in vitro or left uninfected. Panels show representative light microscopic and immunofluorescence images. Bar = 50 μM.
(TIF)

S4 Fig CagA regulates ULF, SIVA1, and p19ARF proteins in the murine stomach. (A) Expression of p19ARF, ULF, and SIVA1 proteins were assessed in the murine stomach using Western blotting. Gastric specimens were harvested from mice infected with wild type *H*. *pylori* strain PMSS1 or its *cagE-* isogenic mutant. Three randomly selected mice in each group were analyzed. Uninfected mice served as a control. The graph panels show the densitometric analysis of the corresponding Western blots. Expression of the corresponding proteins in the stomachs of uninfected animals were arbitrarily set at 1. Statistical analysis was performed using an unpaired 2-tailed t-test; ns, statistically not significant; *p < 0.05; **p < 0.01; ***p < 0.001. (B) Representative images show the murine stomachs from p19ARF null C57BL/6 mice infected with *H*. *pylori* strain PMSS1 or left uninfected for 8 months. (C) A representative H&E image of gastric high-grade dysplasia in ARF KO mouse infected with *H*. *pylori* strain PMSS1 for 8 months.
(TIF)

S5 Fig Analyses of CagA binding. (A) SNU1 cells were co-cultured with the indicated *H*. *pylori* isogenic mutants for 6 hours and analyzed for CagA tyrosine phosphorylation (p-CagA). (B) AGS cells were transfected with plasmids expressing wild type CagA or its phosphorylation-deficient mutant (PR CagA) and analyzed for binding of CagA to MKRN1 using co-immunoprecipitation. Binding of CagA to ULF was served as a positive control. (C) FLAG-tagged ULF and SIVA1 deletion mutants as well as wild type controls were transfected into AGS cells that were co-cultured with *H*. *pylori* strain 7.13 for 30 minutes (for SIVA1 IP) or 3 hours (for ULF IP). Cell lysates were immunoprecipitated with FLAG tag antibodies and analyzed for CagA binding using Western blotting. Gel loading was normalized for expression of the corresponding mutants. Each experiment was repeated three times (n = 3). Upper panel shows a scheme depicting SIVA1 and ULF deletions and domain structures. All mutants express the FLAG tag (yellow boxes).
(TIF)
