# Supplementary figures and images for: Helicobacter pylori pathogen inhibits cellular responses to oncogenic stress and apoptosis
Source: PLoS Pathog. 2022 Jun 29;18(6):e1010628. doi: 10.1371/journal.ppat.1010628 (PMC9242521; doi:10.1371/journal.ppat.1010628)

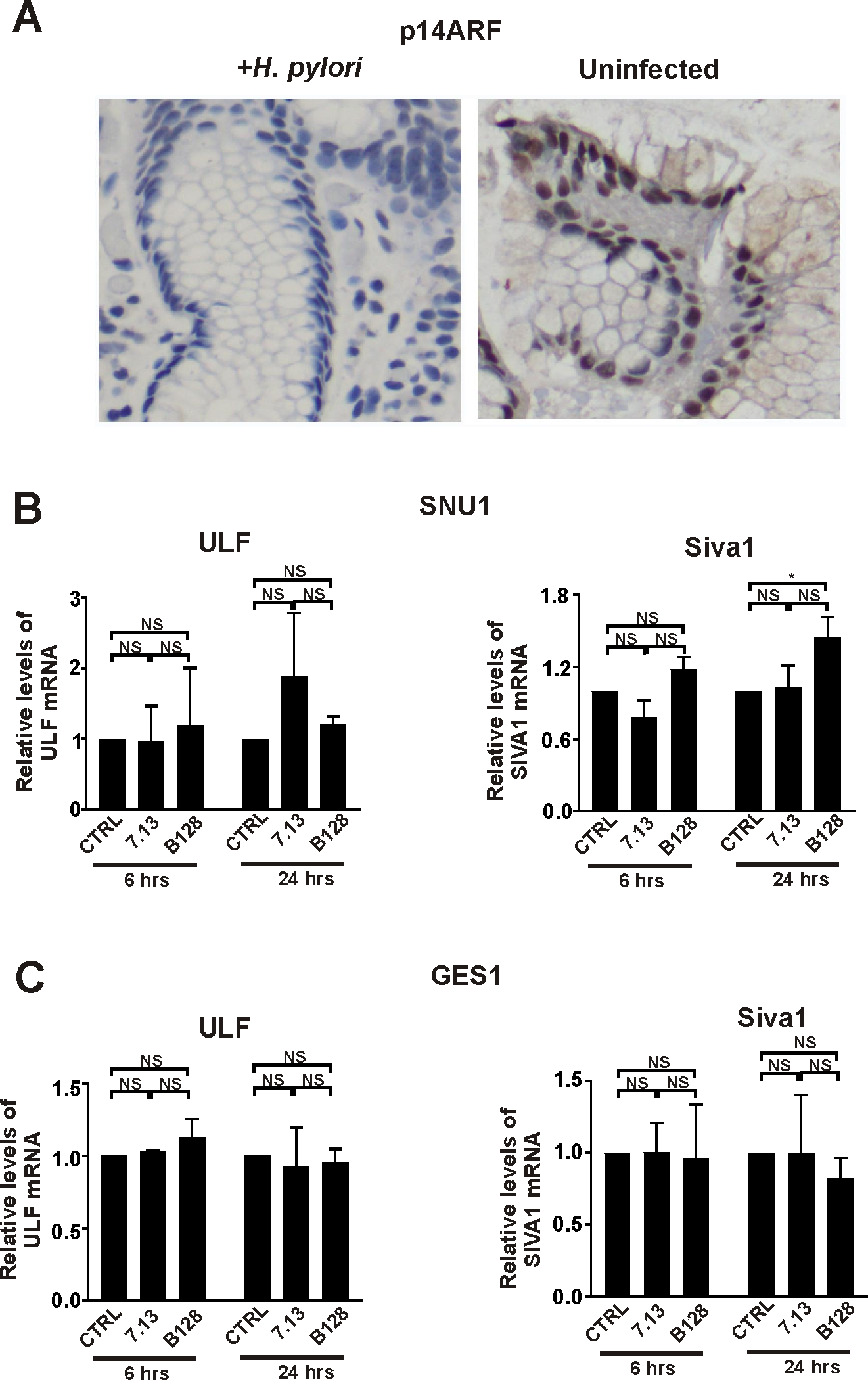

Supplement: S1 Fig — (A) Representative images showing IHC staining of p14ARF protein in the human stomachs of uninfected and H. pylori-infected subjects. (B) qPCR analysis of ULF and SIVA1 mRNA in SNU1 cells co-cultured with H. pylori strains 7.13 or B128 for the indicated time. (C) The same as (B) but GES1 cells were analyzed. (TIF) [file ppat.1010628.s001.TIF]

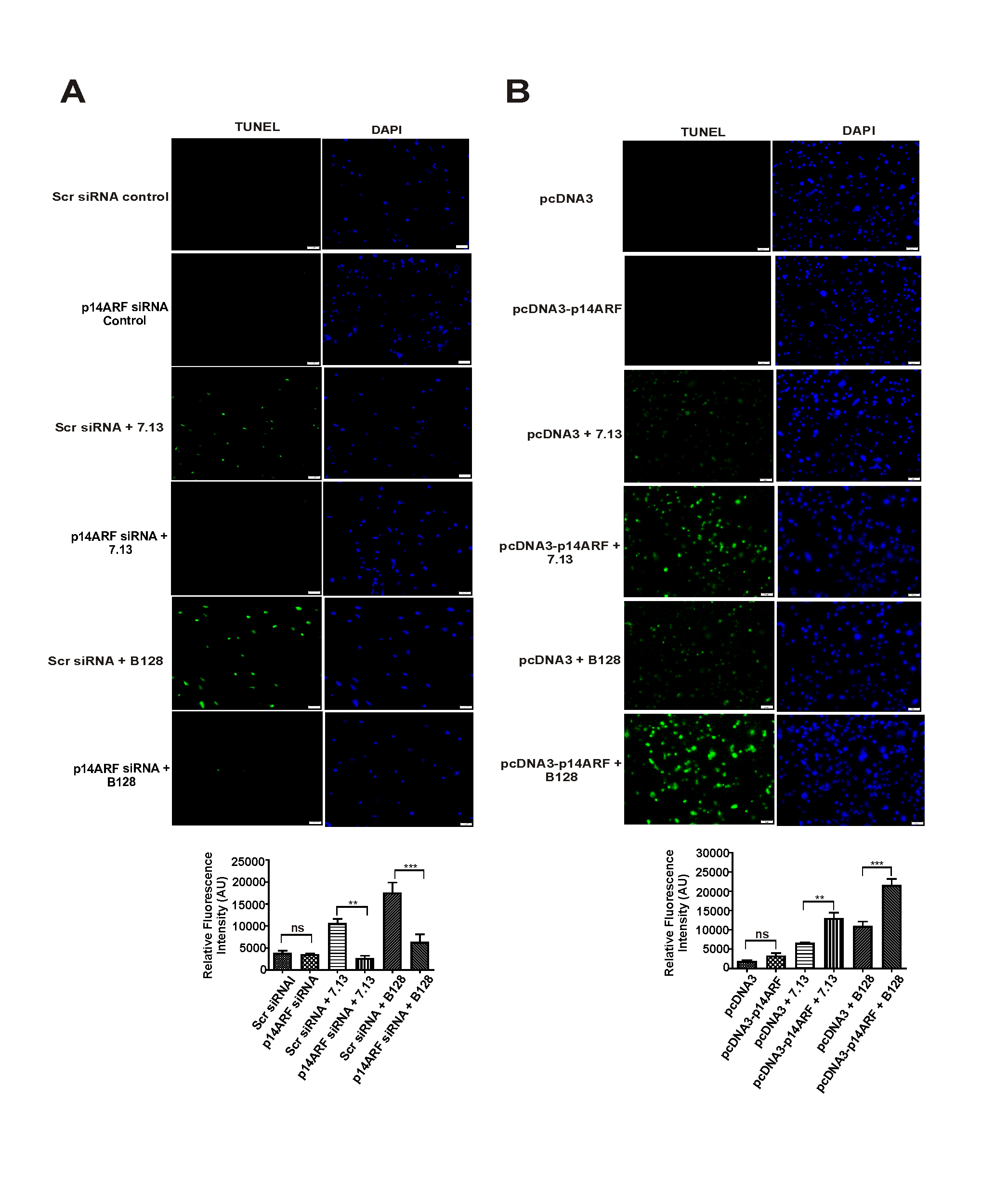

Supplement: S2 Fig — (A) TUNEL analysis was performed in SNU1 cells transfected with p14ARF siRNA or control scrambled siRNA and then either co-cultured with H. pylori strains 7.13 or B128 for 18 hours or left uninfected. (B) The same as (A), but AGS cells were transfected with pcDNA3-p14ARF expression plasmid or empty pcDNA3 vector. Each experiment was carried out three times (n = 3). Bottom panels show quantification of TUNEL staining. Data were analyzed using one-way ANOVA. Data are displayed as mean ± SD. (TIF) [file ppat.1010628.s002.TIF]

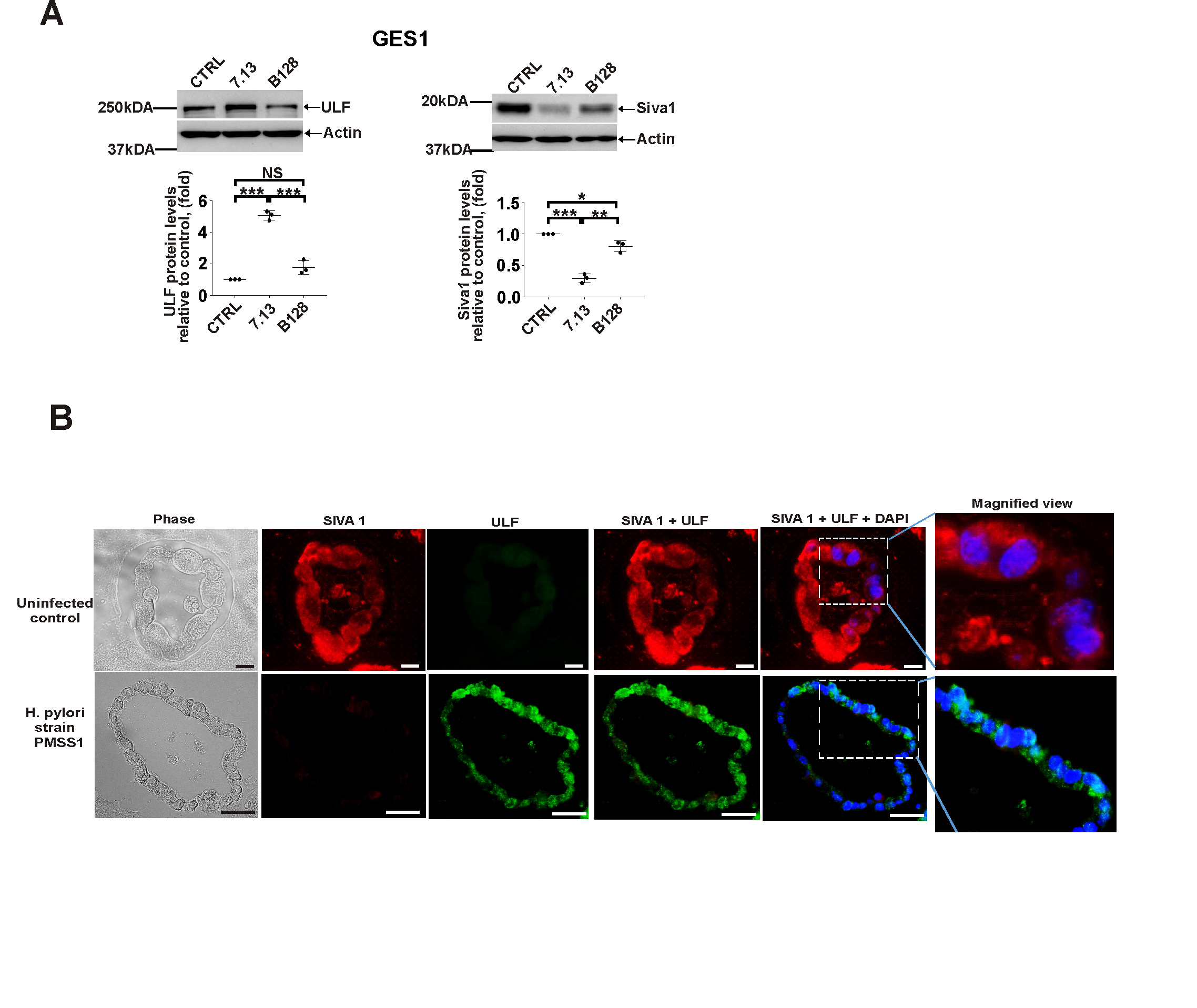

Supplement: S3 Fig — (A) Expression analysis of ULF and SIVA1 proteins in GES1 cells. GES1 cells were co-cultured with H. pylori strains 7.13 or B128 for 6 hours and analyzed for expression of ULF and SIVA1 proteins by Western blotting. (B) Analyses of expression of SIVA1 and ULF proteins in gastric organoids derived from the antropyloric region of the murine stomach that were infected with H. pylori strain PMSS1 in vitro or left uninfected. Panels show representative light microscopic and immunofluorescence images. Bar = 50 μM. (TIF) [file ppat.1010628.s003.TIF]

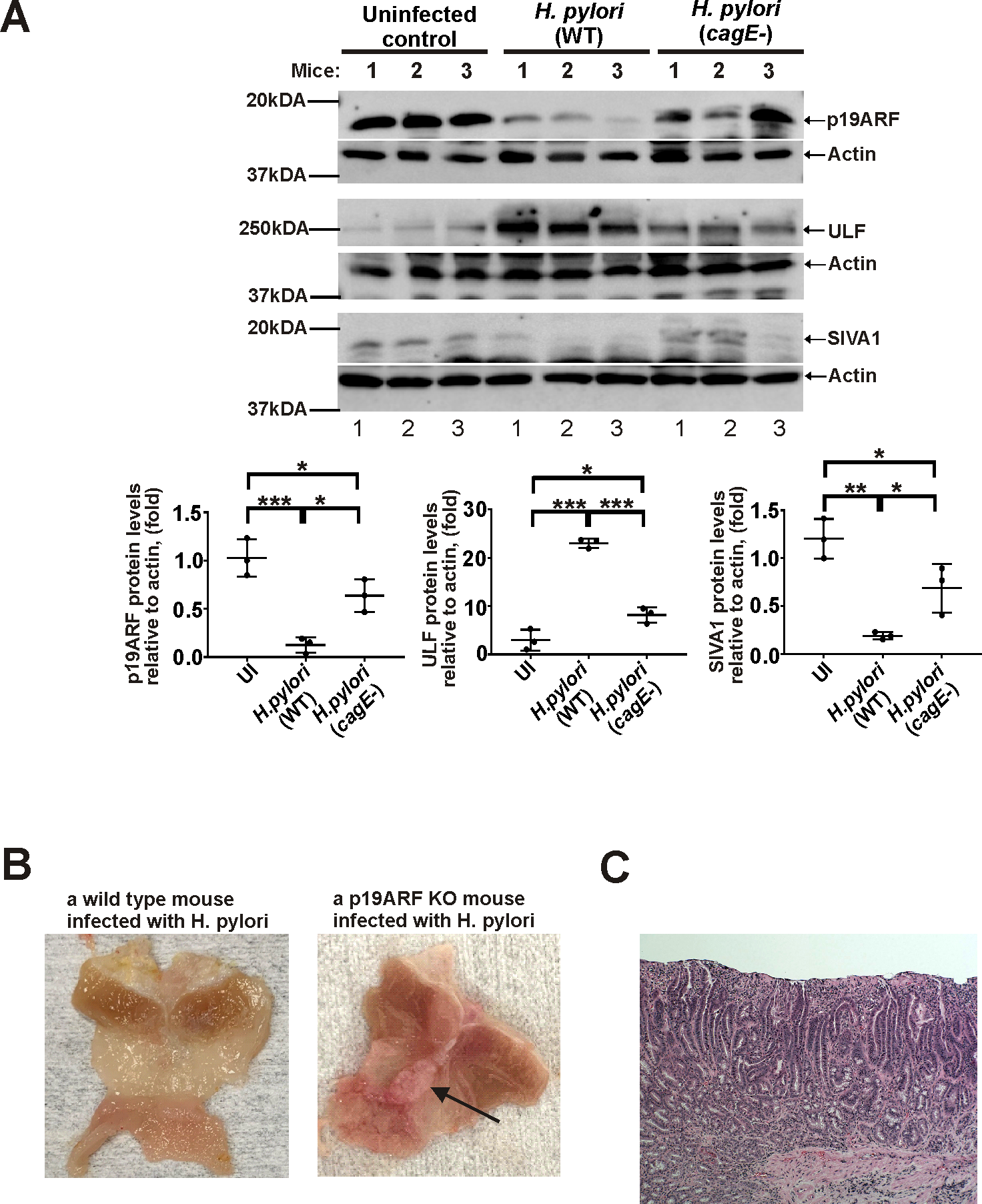

Supplement: S4 Fig — (A) Expression of p19ARF, ULF, and SIVA1 proteins were assessed in the murine stomach using Western blotting. Gastric specimens were harvested from mice infected with wild type H. pylori strain PMSS1 or its cagE- isogenic mutant. Three randomly selected mice in each group were analyzed. Uninfected mice served as a control. The graph panels show the densitometric analysis of the corresponding Western blots. Expression of the corresponding proteins in the stomachs of uninfected animals were arbitrarily set at 1. Statistical analysis was performed using an unpaired 2-tailed t-test; ns, statistically not significant; *p < 0.05; **p < 0.01; ***p < 0.001. (B) Representative images show the murine stomachs from p19ARF null C57BL/6 mice infected with H. pylori strain PMSS1 or left uninfected for 8 months. (C) A representative H&E image of gastric high-grade dysplasia in ARF KO mouse infected with H. pylori strain PMSS1 for 8 months. (TIF) [file ppat.1010628.s004.TIF]

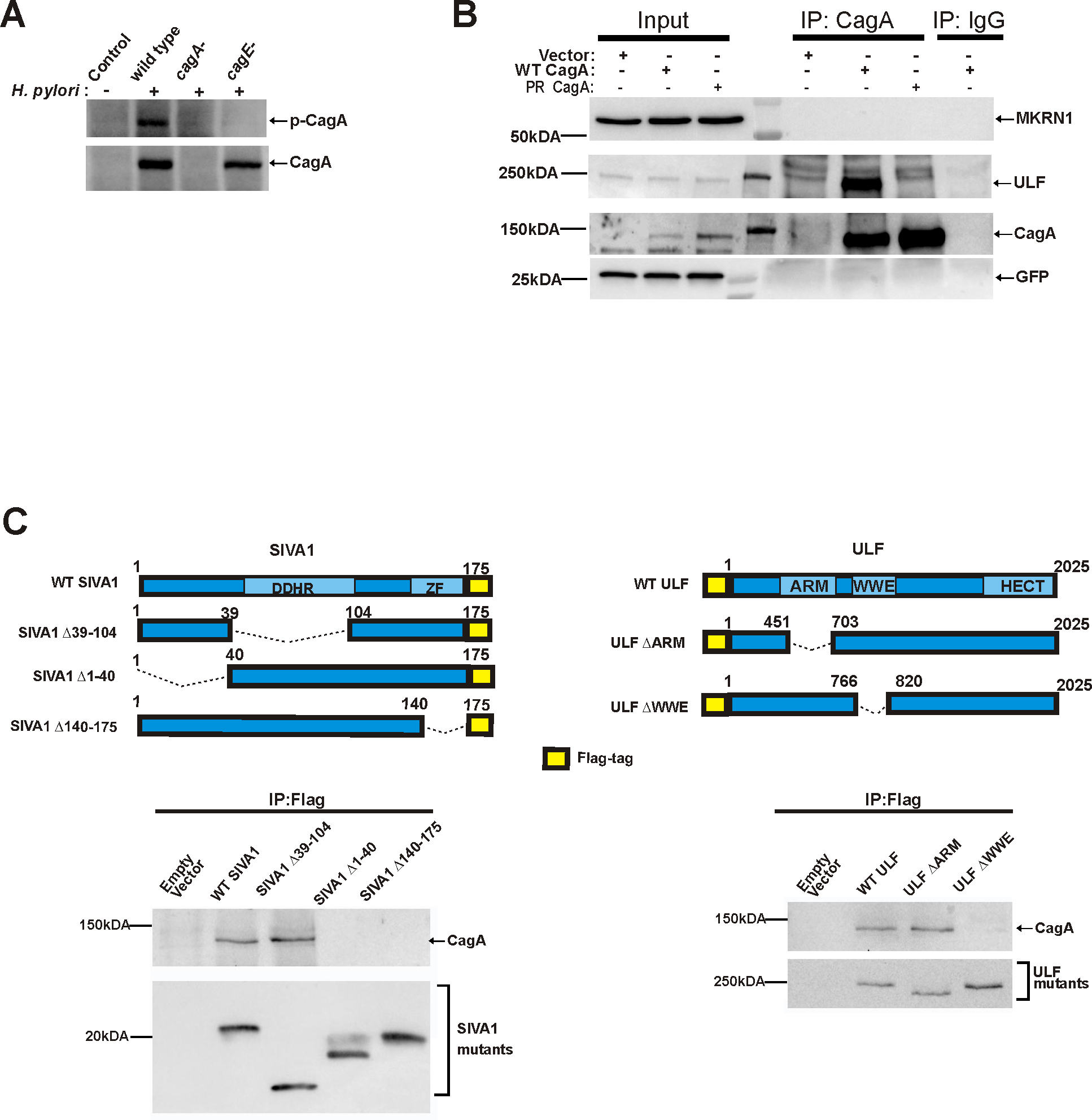

Supplement: S5 Fig — (A) SNU1 cells were co-cultured with the indicated H. pylori isogenic mutants for 6 hours and analyzed for CagA tyrosine phosphorylation (p-CagA). (B) AGS cells were transfected with plasmids expressing wild type CagA or its phosphorylation-deficient mutant (PR CagA) and analyzed for binding of CagA to MKRN1 using co-immunoprecipitation. Binding of CagA to ULF was served as a positive control. (C) FLAG-tagged ULF and SIVA1 deletion mutants as well as wild type controls were transfected into AGS cells that were co-cultured with H. pylori strain 7.13 for 30 minutes (for SIVA1 IP) or 3 hours (for ULF IP). Cell lysates were immunoprecipitated with FLAG tag antibodies and analyzed for CagA binding using Western blotting. Gel loading was normalized for expression of the corresponding mutants. Each experiment was repeated three times (n = 3). Upper panel shows a scheme depicting SIVA1 and ULF deletions and domain structures. All mutants express the FLAG tag (yellow boxes). (TIF) [file ppat.1010628.s005.TIF]
